# Supplementary figures and images for: Retrospective evaluation of a novel ultrasound-based imaging analysis software for predicting radiofrequency ablation areas
Source: PLoS One. 2025 Jan 17;20(1):e0317469. doi: 10.1371/journal.pone.0317469 (PMC11741625; doi:10.1371/journal.pone.0317469)

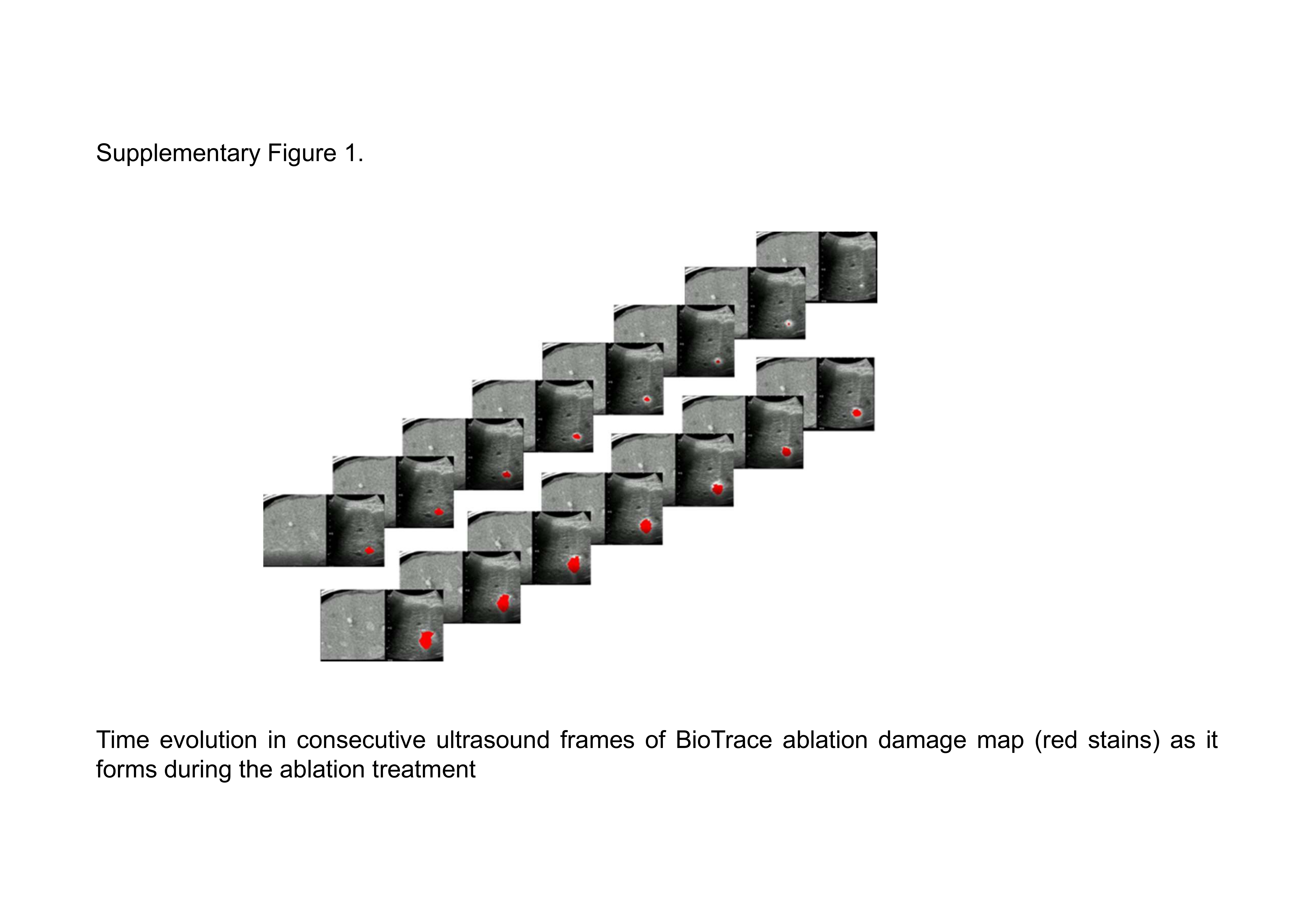

Supplement: S1 Fig — (TIF) [file pone.0317469.s001.tif]

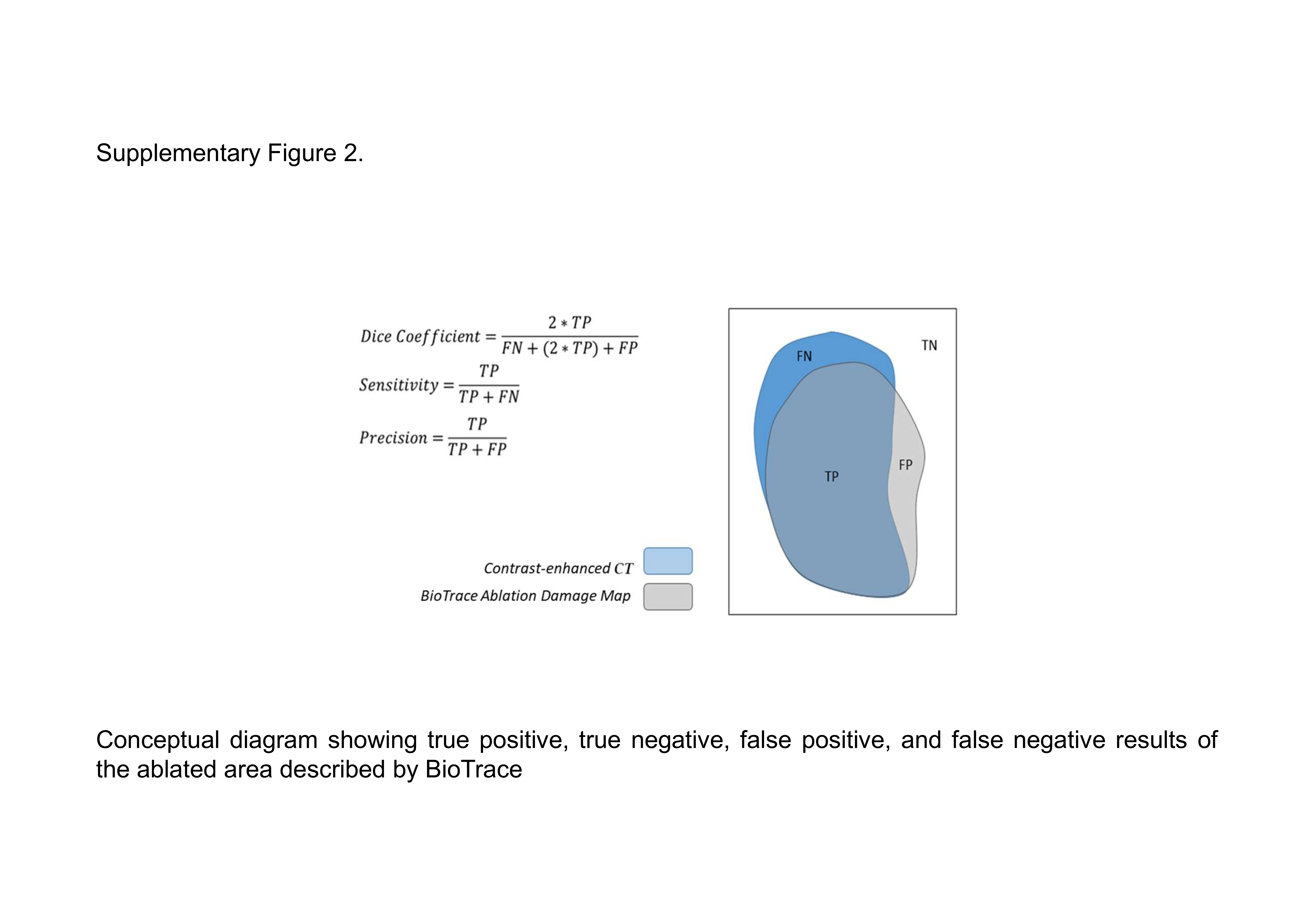

Supplement: S2 Fig — (TIF) [file pone.0317469.s002.tif]

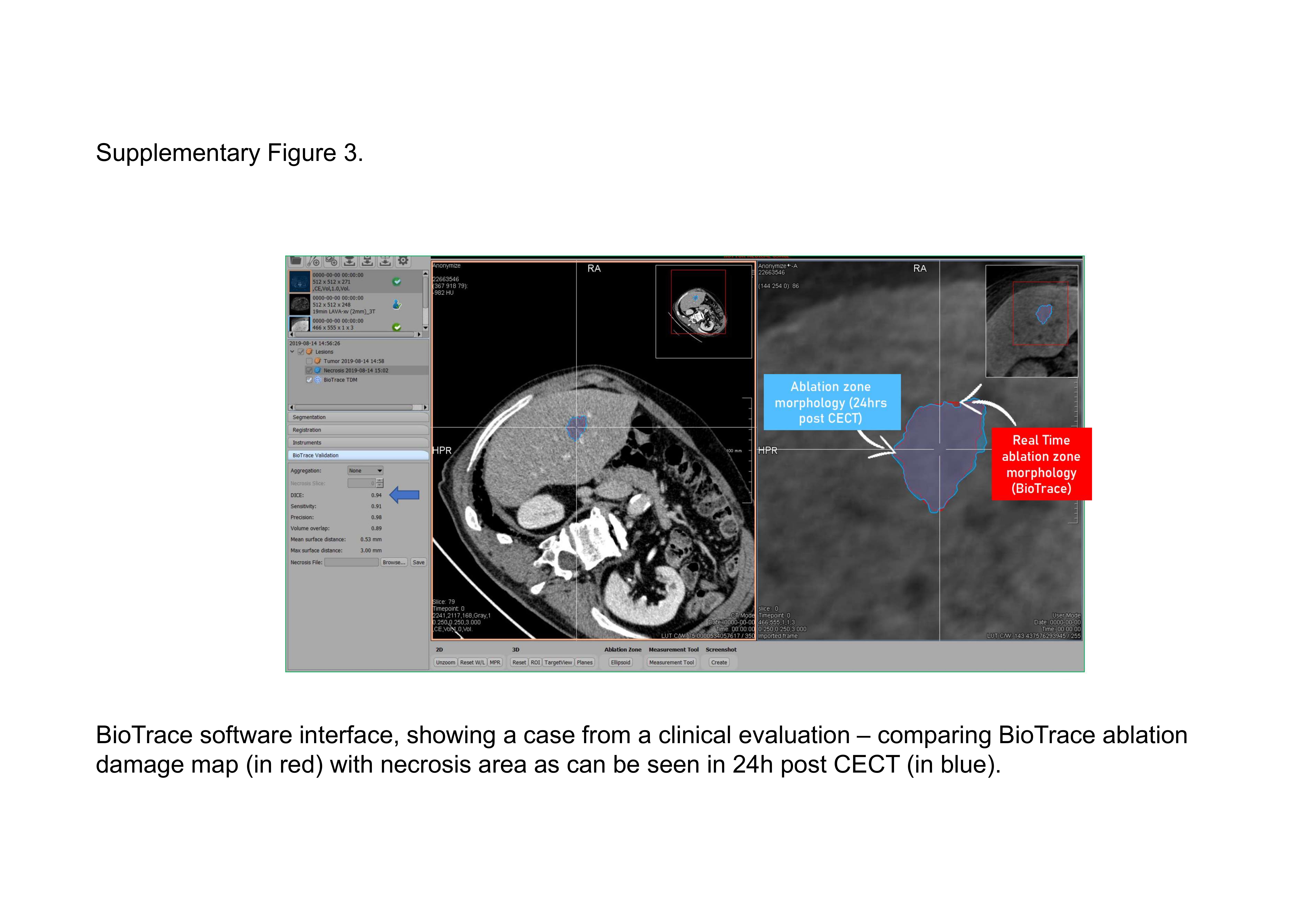

Supplement: S3 Fig — (TIF) [file pone.0317469.s003.tif]
