# Supplementary material for: Retrospective evaluation of a novel ultrasound-based imaging analysis software for predicting radiofrequency ablation areas
Source: PLoS One. 2025 Jan 17;20(1):e0317469. doi: 10.1371/journal.pone.0317469 (PMC11741625; doi:10.1371/journal.pone.0317469)
Supplement: S1 File — (DOCX) [file pone.0317469.s004.docx]

**Supporting information**

| Ablated  tumor  number | Tumor size (cm) | Tumor location | BioTrace surface  (mm^2^) | Radiologists  surface  (mm^2^) | Sex | Age | Hybrid maps comprising the necrosis area as demonstrated by BioTrace (red) and the radiologists (blue) |
| --- | --- | --- | --- | --- | --- | --- | --- |
| 1. | 0.8 | 6 | 477.66 | 470.67 | Male | 64 | 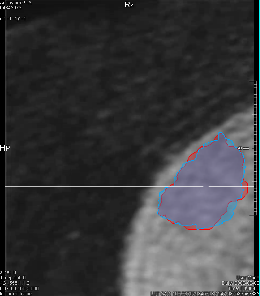 |
| 2. | 1.5 | 7 | 555.7 | 556.85 | Female | 81 | 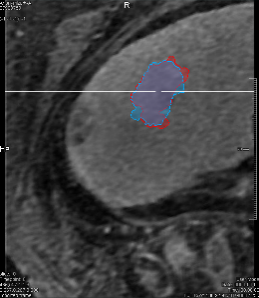 |
| 3. | 1.1 | 5 | 687.91 | 687.1 | Male | 74 | 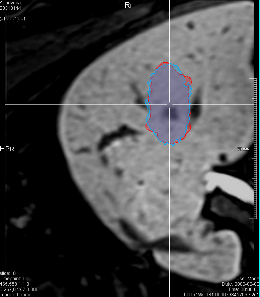 |
| 4. | 1.2 | 8 | 475.08 | 536.71 | Male | 62 | 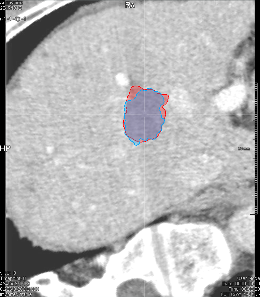 |
| 5. | 1.1 | 7 | 423.36 | 362.47 | Male | 62 | 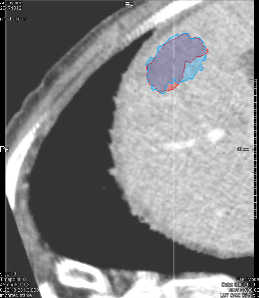 |
| 6. | 1.5 | 3 | 874.05 | 871.99 | Male | 72 | 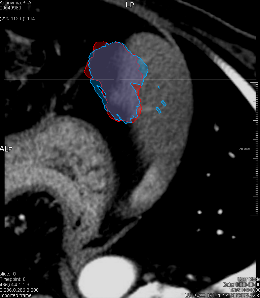 |
| 7. | 1.7 | 7 | 404.34 | 362.62 | Male | 72 | 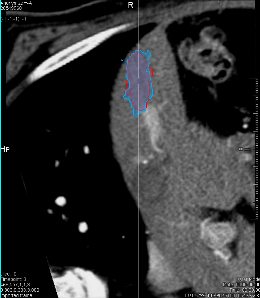 |
| 8. | 1.7 | 6 | 964.98 | 1032.03 | Male | 64 | 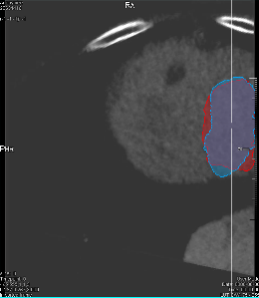 |
| 9. | 1.7 | 8 | 617.86 | 628.99 | Female | 77 | 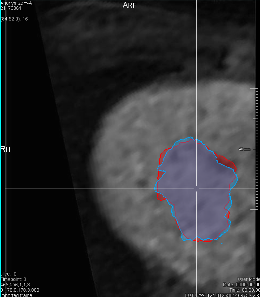 |
| 10. | 1.0 | 8 | 282.36 | 307.16 | Female | 76 | 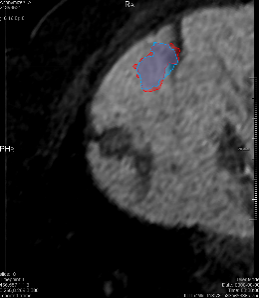 |
| 11. | 1.0 | 6 | 248.09 | 259.17 | Male | 79 | 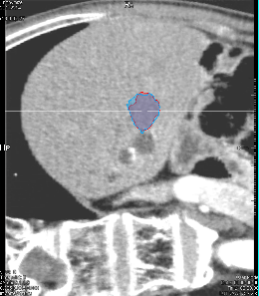 |
| 12. | 1.2 | 5 | 362.6 | 326.69 | Male | 86 | 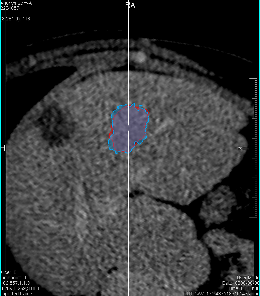 |
| 13. | 0.8 | 5 | 333.11 | 338.43 | Male | 74 | 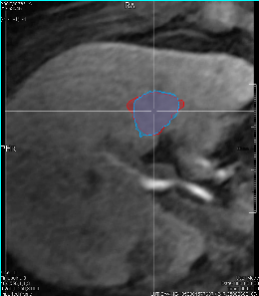 |
| 14. | 0.8 | 6 | 268.69 | 253.06 | Male | 74 | 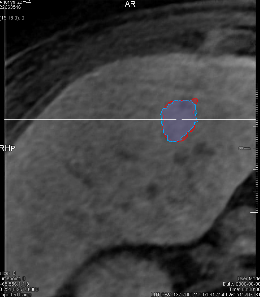 |
| 15. | 1.3 | 7 | 448.92 | 498.74 | Male | 74 | 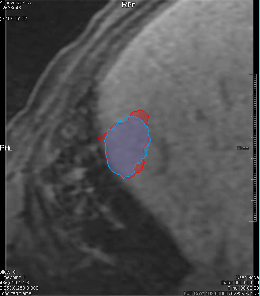 |
| 16. | 0.7 | 8 | 255.36 | 296.56 | Female | 65 | 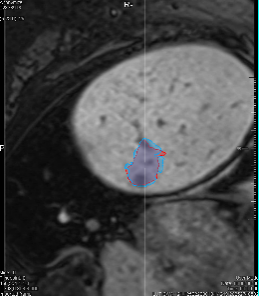 |
| 17. | 0.9 | 7 | 777.05 | 723.26 | Female | 65 | 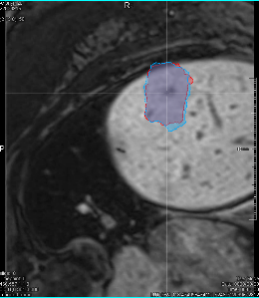 |
| 18. | 1.2 | 8 | 599.99 | 524.73 | Female | 65 | 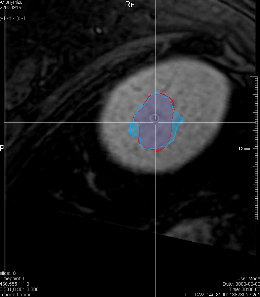 |
| 19. | 2.0 | 3 | 440.57 | 439.8 | Female | 64 | 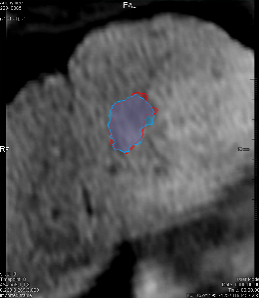 |
| 20. | 1.8 | 6 | 726.21 | 773.61 | Female | 64 | 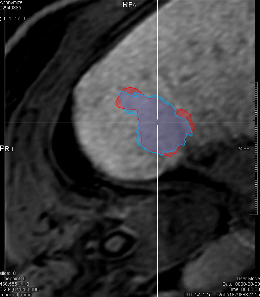 |
