# Supplementary material for: Retrospective evaluation of a novel ultrasound-based imaging analysis software for predicting radiofrequency ablation areas
Source: PLoS One. 2025 Jan 17;20(1):e0317469. doi: 10.1371/journal.pone.0317469 (PMC11741625; doi:10.1371/journal.pone.0317469)
Supplement: Attachment — Submitted filename: Response to Reviewers letter20241227.docx [file pone.0317469.s006.docx]

**Point-by-point responses to the reviewers’ comments:**

**Reviewer 3**

1. In the current study, you compared performance of BTM (pre ablation prediction) with CECT 24 hours after ablation, please mention this in a clear statement in methodology section. In addition, BTM system is not activated during the procedure in the current study. So I think the title should be modified express the work in this study.

Please revise the following:
((A Novel Real-Time Ultrasound-Based Imaging Analysis Software for Monitoring Radiofrequency Ablation and Visualizing Ablation Areas))
In the current study you said in page 15” Although the current system has been designed for real-time monitoring, it was not activated during the procedure in this study.
Page 14” It provides comparable performance in assessing the treatment response of radiofrequency ablation (RFA).”
Unfortunately, the study design is not compatible with prospective observational study! You compared performance of BTM with CECT 24 hours after ablation in the same group.

[Response]

We agree that the methodology and title should be clarified to better reflect the nature of our study.

We modified the title of our manuscript as follows

Page 1, Title

Retrospective Evaluation of a Novel Ultrasound-Based Imaging Analysis Software for Predicting Radiofrequency Ablation Areas

We have added the following statement in the Method section under "Performance evaluation of BioTrace and statistical analysis” as follows.

Page 7, “Patients and procedures”

This study is a retrospective analysis of prospectively collected ultrasound imaging data, aiming to evaluate the predictive capabilities of the BioTrace system.

Page 9, “Performance evaluation of BioTrace and statistical analysis”

In this study, we retrospectively analyzed the ablation areas by comparing the BioTrace prediction generated from ultrasound imaging data collected during the RFA procedure with the actual ablation zones confirmed by CECT obtained 24 hours post-treatment, which is considered the gold standard. While the BTM system is designed for real-time monitoring, it was not activated during the procedures in this study. Instead, we retrospectively applied the BioTrace algorithm to the prospectively collected ultrasound imaging videos to generate the ablation prediction maps.

Regarding the description on page 14 and page 15 (in the pre revise version), we have addressed the issues and revised the manuscript as follows.

Page 15, first paragraph

Additionally, the BioTrace system demonstrated comparable performance to CT in assessing ablation zones in this retrospective analysis, suggesting potential advantages for future intra-operative monitoring applications.

Page 16, second paragraph

While the BioTrace system is designed for real-time monitoring, this validation study focused on retrospective analysis of prospectively collected ultrasound imaging data. The real-time monitoring capabilities will be evaluated in future prospective clinical trials.

2. What is/are the extra benefit/s expected from BTM in contrast to ablation under the guidance of contrast-enhanced ultrasonography? Both are ultrasound based and real time technique!

[Response]

We appreciated the reviewer’s important question regarding the advantages of BioTrace (BTM) compared to contrast-enhanced ultrasonography. The BioTrace system offers several distinct advantages over contrast-enhanced ultrasonography (CEUS). First, it minimizes the resources required for contrast agent preparation and administration. Second, unlike CEUS which requires low acoustic pressure to maintain microbubble stability, BioTrace can utilize optimal ultrasound settings to maintain high-quality B-mode imaging throughout the procedure. This allows for better visualization of the target area and surrounding structures during ablation.

We have added the current information to our manuscript as follows.

Page 14, third paragraph

The BioTrace system offers several distinct advantages over contrast-enhanced ultrasonography. First, it minimizes the resources required for contrast agent preparation and administration. Second, unlike CEUS which requires low acoustic pressure to maintain microbubble stability, BioTrace can utilize optimal ultrasound settings to maintain high-quality B-mode imaging throughout the procedure. This allows for better visualization of the target area and surrounding structures during ablation.

3. Page 8, you mentioned “The dynamics of microbubbles, reflecting the tissue’s response to heat, varies according to the applied temperature” I think there are multiple variables other than temperature should be considered e.g., biological nature of lesion, pathological background of liver. I mean HCC on top of chronic hepatitis, cirrhosis or viral VS metabolic liver disease, fibrotic load within the liver,……

[Response]

We understand the reviewer’s comment. We have revised the manuscript to better reflect the complexity of these factors influencing the ablation process as follows.

Page 8, second paragraph

The dynamics of microbubbles reflect a complex interaction of multiple variables during thermal ablation. While temperature is a primary factor, the microbubble behavior is also influenced by: (1) the biological nature of the lesion, (2) the underlying pathological condition of the liver (such as chronic hepatitis, cirrhosis, or metabolic liver disease), (3) the degree of liver fibrosis, (4) local tissue perfusion characteristics, and (5) the applied temperature. These variables collectively affect the formation and the dynamic behavior of microbubbles during thermal ablation.

We also added the text to emphasize the need for further investigation across different liver conditions to better understand these complex interactions as follows.

Page 16, second paragraph

Furthermore, the influence of various liver pathologies and tissue characteristics on microbubble dynamics requires additional investigation. Future studies should evaluate the system's performance across different pathological conditions, including chronic hepatitis, cirrhosis, and varying degrees of liver fibrosis, to better understand how these factors affect the accuracy of ablation zone prediction.
