# Supplementary material for: Retrospective evaluation of a novel ultrasound-based imaging analysis software for predicting radiofrequency ablation areas
Source: PLoS One. 2025 Jan 17;20(1):e0317469. doi: 10.1371/journal.pone.0317469 (PMC11741625; doi:10.1371/journal.pone.0317469)
Supplement: Attachment — Submitted filename: Reviewer comments and recommendations.docx [file pone.0317469.s005.docx]

The authors of this manuscript presented a novel Real-Time Ultrasound-based imaging analysis software for monitoring radiofrequency ablation and visualizing ablation areas. This title is worthy of investigation and the study is well organized and well structured. However, some comments are mentioned below that need more clarification, explanation, or modification.

1. The abstract of the study is better to be structured.
2. All the abbreviations in the manuscript like CT, MRI, etc. should first be mentioned in their complete forms followed by their abbreviated forms.
3. Please mention the type of study in the method section.
4. How many times each experiment was applied? Please explain.
5. Was the sample size enough to reach an accurate and robust result? Please explain.
6. Did your study involve any sort of follow-ups or not?
7. Please mention exactly the control groups that you have utilized in your study.

Wish you luck and prosperity in revising the manuscript.
